# Identification of Ubiquitin-Related Gene-Pair Signatures for Predicting Tumor Microenvironment Infiltration and Drug Sensitivity of Lung Adenocarcinoma

**DOI:** 10.3390/cancers14143478

**Published:** 2022-07-18

**Authors:** Yumei Li, Lanfen An, Zhe Jia, Jingxia Li, E Zhou, Feng Wu, Zhengrong Yin, Wei Geng, Tingting Liao, Wenjing Xiao, Jingjing Deng, Wenjuan Chen, Minglei Li, Yang Jin

**Affiliations:** 1 NHC Key Laboratory of Pulmonary Diseases, Hubei Province Engineering Research Center for Tumor-Targeted Biochemotherapy, Department of Respiratory and Critical Care Medicine, Union Hospital, Tongji Medical College, Huazhong University of Science and Technology, Wuhan 430022, China; yumei_li@hust.edu.cn (Y.L.); m202175781@hust.edu.cn (Z.J.); m202175768@hust.edu.cn (J.L.); zhoue@hust.edu.cn (E.Z.); 2011xh0839@hust.edu.cn (F.W.); yinzhengrong@hust.edu.cn (Z.Y.); wguh116@hust.edu.cn (W.G.); d201881381@hust.edu.cn (T.L.); d201981508@hust.edu.cn (W.X.); m202075773@hust.edu.cn (J.D.); wenjuan_chen@hust.edu.cn (W.C.); d202181823@hust.edu.cn (M.L.); 2 Department of Obstetrics and Gynecology, Union Hospital, Tongji Medical College, Huazhong University of Science and Technology, Wuhan 430022, China; alf9105@hust.edu.cn; 3 Clinical Center of Reproductive Medicine, The First Affiliated Hospital of USTC, Division of Life Science and Medicine, University of Science and Technology of China, Hefei 230001, China

**Keywords:** ubiquitin, ubiquitin-related gene pair, tumor microenvironment, immune infiltration, tumor mutation burden, drug sensitivity, lung adenocarcinoma

## Abstract

**Simple Summary:**

Lung adenocarcinoma (LUAD) has a high mortality and incidence rate. The therapeutic efficacy of LUAD varies with the individual heterogeneity of the tumor microenvironment (TME). It is necessary to explore more biomarkers and targets to improve the prognosis of patients. Ubiquitination pathways are involved in the biological process of regulating the anti-tumor immunity of immune cells and immunosuppression of tumor cells in the TME of patients. In this study, we clarified the characteristics of ubiquitin-related gene pairs (UbRGPs) and identified the relationship between the status of the TME and UbRGPs of patients with LUAD. A prognostic signature based on six UbRGPs was established, which performed well in predicting the immune infiltration and tumor mutation burden (TMB) in the TME and the response of LUAD to immuno-, chemo-, and targeted therapy. In conclusion, the UbRGPs signature is an independent prognostic indicator and has great potential in assisting the clinical therapy for patients with LUAD.

**Abstract:**

Lung adenocarcinoma (LUAD) is a common pathological type of lung cancer worldwide, and new biomarkers are urgently required to guide more effective individualized therapy for patients. Ubiquitin-related genes (UbRGs) partially participate in the initiation and progression of lung cancer. In this study, we used ubiquitin-related gene pairs (UbRGPs) in tumor tissues to access the function of UbRGs in overall survival, immunocyte infiltration, and tumor mutation burden (TMB) of patients with LUAD from The Cancer Genome Atlas (TCGA) database. In addition, we constructed a prognostic signature based on six UbRGPs and evaluated its performance in an internal (TCGA testing set) and an external validation set (GSE13213). The prognostic signature revealed that risk scores were negatively correlated with the overall survival, immunocyte infiltration, and expression of immune checkpoint inhibitor-related genes and positively correlated with the TMB. Patients in the high-risk group showed higher sensitivity to partially targeted and chemotherapeutic drugs than those in the low-risk group. This study contributes to the understanding of the characteristics of UbRGPs in LUAD and provides guidance for effective immuno-, chemo-, and targeted therapy.

## 1. Introduction

Lung adenocarcinoma (LUAD) is the most common histological lung cancer, and it is the leading cause of cancer-related deaths worldwide [1,2]. The treatment for LUAD includes surgery and radio-, chemo-, immuno-, targeted, and palliative therapy [3]. The therapeutic effect in most patients with LUAD remains unsatisfactory. Therefore, newly emerging biomarkers are urgently required to guide the therapeutic options and improve the clinical outcomes of patients with LUAD.

Ubiquitin and ubiquitin-like (UB/UBL) modifications are regulated by ubiquitin-activating enzymes (E1s), ubiquitin-conjugating enzymes (E2s), ubiquitin-protein ligases (E3s), deubiquitinating enzymes (DUBs), ubiquitin/ubiquitin-like binding domains (UBDs), and ubiquitin-like domains (ULDs) [4]. Ubiquitin pathways perform various physiological functions in different types of immune and cancer cells [5]. Ge et al. [6] summarized the partial functions of the ubiquitin pathway in cancers as follows: first, the ubiquitin pathway is related to the oncogenesis and progression of cancers, including the cell cycle, p53 activation, DNA damage repair, and apoptosis [6]. Second, targeting the ubiquitin pathway has become a promising treatment strategy for patients with cancer [6]. Yang et al. identified a group of ubiquitin-related genes as the criteria to classify the molecular subtypes and stratify the risk of patients [7]. The Food and Drug Administration (FDA) has permitted several drugs targeting ubiquitin-related pathways for cancer treatment [8,9]. The combination of bortezomib with existing chemotherapeutic agents or targeted therapy for non-small cell lung cancer (NSCLC) has achieved success in phase I and II clinical studies [10].

Previous studies have shown that several ubiquitin-related genes (UbRGs) participated in the initiation and development of lung cancer [11,12]. Ubiquitin binding enzyme E2 (UBE2) H promoted metastasis and was negatively correlated with prognosis for LUAD [13]. The expression of *UBE2S* was enhanced in tumor samples and correlated with poor clinical outcomes in LUAD [14]. The overexpressed *UBE2C* promoted the biological behaviors of lung cancer and was associated with the poor clinical outcome of LUAD [15,16]. The decreased level of FBXW7 is correlated with the bad survival and chemo-resistance of patients with lung cancer [17,18,19]. Thus, ubiquitin-related pathways are associated with the tumorigenesis, development, and survival of patients with lung cancer. Moreover, various inhibitors targeting UbRGs are an emerging choice for clinical practice in lung cancer therapy [20]. Nevertheless, the function of UbRGs in LUAD has not been systematically investigated.

In this study, we clarified the characteristics of ubiquitin-related gene pairs (UbRGPs) and identified the diversity of the tumor microenvironment (TME) in different subtypes of patients with LUAD from The Cancer Genome Atlas (TCGA) cohort. A prognostic signature based on six UbRGPs was established, and its performance in predicting the immune infiltration and tumor mutation burden (TMB) in TME was further analyzed. Finally, we used this signature to evaluate the clinical prognosis and response of LUAD to immuno-, chemo-, and targeted therapy.

## 2. Materials and Methods

### 2.1. Multi-Omics Data Extraction and Patient Information Precondition

**Figure 1:**
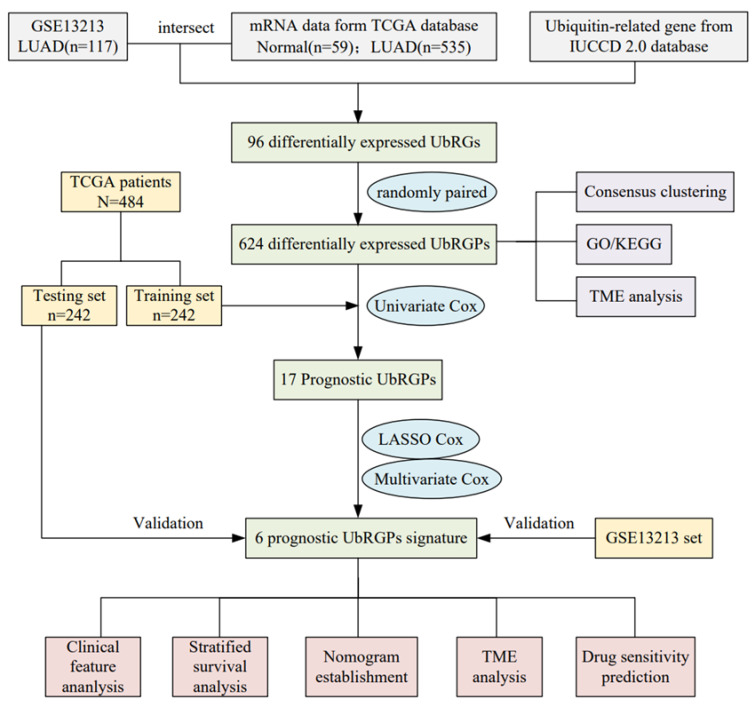
The flow chart of the study.

The detailed workflow of our study is shown as a chart (Figure 1). The transcriptome profiles, somatic mutation data, and clinical characteristics of patients with LUAD were obtained from the TCGA database. Patients without clinicopathological data or overall survival (OS) times were excluded from this study. Further, we randomly separated the 484 patients with LUAD from the TCGA cohort into two groups using the “caret” package, consisting of testing and training sets. The set of training, including 242 patients, was used to construct the prognostic signature, and the set of testing was used for internal validation. For external validation, matrix files, basic clinical, and survival information of an independent dataset GSE13213 (*n* = 117) were extracted from the Gene Expression Omnibus (GEO) database. The clinicopathological data of the cohorts are shown in Table 1, indicating that the training and testing cohorts were homogeneous.

### 2.2. Construction of Differentially Expressed Ubiquitin-Related Gene Pairs (UbRGPs)

A total of 1366 UbRGs were retrieved from the iUUCD 2.0 database [4]. The available mRNA expression profiles of 633 ubiquitin-related genes were mined from the GSE13213 datasets and the TCGA database. And we determined the differentially expressed UbRGs between normal and tumor samples based on the criteria of false discovery rate (FDR) <0.05 and |log2 Fold change (FC)| > 1. The differentially expressed UbRGs from the TCGA database were intersected with GSE13213 datasets, and we selected 96 UbRGs expressed in tumor tissues with a cut-off criterion of median absolute deviation >0.5.

The gene expression level of the differentially expressed UbRGs in each tumor tissue was randomly pairwise compared to generate a score for each UbRGP [21]. When the UbRG1 expression was higher than that of UbRG 2, the UbRGP score was 1; otherwise, the UbRGP score was 0, and a UbRGP expression matrix of 0 or 1 was constructed [21]. Some UbRGPs with constant values (the frequency of 0 or 1 is more than 80% or less than 20%) were removed, which provided no discriminative information about the patient survival [22]. Finally, the differentially expressed UbRGPs (0 or 1 frequency between 20% and 80%) in each dataset were established.

### 2.3. Consensus Clustering

Consensus clustering, an analysis approach to guide the use of clustering algorithms and evaluate the stability of the discovered clusters, is a resampling-based methodology for class discovery and visualization of gene expression data [23]. Based on the differentially expressed matrix of UbRGPs in the tumor tissues of patients with LUAD in the TCGA database, the “ConsensusClusterPlus” R package was used to perform the unsupervised consensus clustering method by setting 8 groups from k = 2 to 9, 80% resampling rate, 50 iterations, and Euclidean distance [24]. The cumulative distribution function (CDF) curves and consensus matrix were used to determine the optimal cluster number [25]. The clusterprofiler package for Gene-Set Enrichment Analysis (GSEA) was used to investigate the signaling pathways involved in the different LUAD clusters from the TCGA database [26]. The chi-square test was used to determine the difference in categorical data.

### 2.4. Immune Infiltration Analysis in the Tumor Microenvironment (TME)

The TME scores, including estimate, stromal, and immune scores for all LUAD samples, were predicted using the “ESTIMATE” package. The “ESTIMATE” package applied the expression of genes to investigate the proportion of cells in samples [27].

To explore immunocyte infiltration in the TME, seven algorithms, including CIBERSORT-ABS [28], CIBERSORT [29], EPIC [30], MCPCOUNTER [31], QUANTISEQ [32], TIMER [33], and xCELL [34], were used to determine the immune infiltration values of the samples. A violin diagram was constructed to show the detailed differences between the two clusters using CIBERSORT. The immunocyte infiltration between the low- and high-risk groups was displayed by heatmap, and the difference was investigated by the Wilcoxon signed-rank test. The correlation coefficient between infiltrating immune cells and the risk score was examined by Spearman correlation analysis and was displayed on the lollipop chart. Comparing the 13 immune-related pathways between the two groups, the immunotherapeutic response of each patient was evaluated using the immunophenoscore (IPS) algorithm, as described previously [35].

### 2.5. Analysis of Somatic Mutations in LUAD

TMB was defined as the number of coding errors of somatic genes, base substitution, and indel mutations per megabase of genome examined [36]. The “Maftools” R package [37], which depends on the somatic variation in mutation annotation format and provides a large number of analysis and visualization modules, was used to analyze the TMB of each sample in the TCGA database. The quantitative analysis of TMB was shown in a boxplot. The 20 most frequently tumor mutated genes were shown in the waterfall plot. Each column represented a patient.

### 2.6. Establishment and Evaluation of UbRGPs-Related Prognostic Signature

Univariate Cox regression analysis was performed to determine the OS-related UbRGPs in the TCGA training cohort when *p* < 0.01. Subsequently, the least absolute shrinkage and selection operator (LASSO) Cox regression algorithm and stepwise multivariate Cox proportional hazard regression analysis were used to construct the UbRGP-based prognostic signature in the training cohort. According to the median risk score calculated from the multivariate Cox model, patients were divided into two groups. The receiver operating characteristic (ROC) curve, survival curve, survival status, and risk score distribution were analyzed.

### 2.7. Correlation Analysis and Clinical Stratification Analysis

The correlation of clinical characteristics with risk scores was analyzed, and a heatmap was plotted for visualization. The differences in risk scores between the low- and high-risk groups were assessed by Wilcoxon signed-rank test, and a box diagram was drawn. The independence of the UbRGPs signature and several clinical features were evaluated by univariate and multivariate Cox regression analysis. To evaluate the clinical applicability of this prognostic signature, samples were stratified into ten subgroups. The survival of patients was analyzed using the log-rank test.

### 2.8. Construction of Calibration Curves and Nomogram

Nomogram was constructed to assess the survival probability of individuals by integrating risk scores and other clinicopathological information by the “RMS” package. The total score of all factors corresponds to the survival probability of each patient [38]. Calibration curves were applied to evaluate the uniformity between actual and predicted survival. In addition, the predicting survival evaluation of the nomogram was assessed by ROC.

### 2.9. Chemotherapeutic Drug Susceptibility Prediction

We also determined the IC_50_ of common targeted therapy and chemotherapy drugs in each sample using pRRophetic [39] to infer drug sensitivity. The guidelines recommend the use of etoposide, paclitaxel, vinorelbine, docetaxel, methotrexate, erlotinib, gefitinib, crizotinib, and alectinib for the treatment of LUAD [2]. The IC_50_ differences between these drugs were analyzed by the Wilcoxon signed-rank test.

### 2.10. Quantitative Real-Time Polymerase Chain Reaction (qRT-PCR)

Ten cancer samples and ten para-cancerous normal lung samples were obtained from patients with LUAD who underwent surgery at the Wuhan Union Hospital (Wuhan, China). Total tissue RNA was extracted using TRIzol reagent (TaKaRa, Japan), and the RNA concentration was measured by the NanoDrop 2000 spectrophotometer (NanoDrop Technologies, Wilmington, DE, USA). RNA (1 µg) was reverse transcribed into cDNA to a final volume of 20 µL using HiScript III RT Supermix (Vazyme, China). qPCR was performed using the AceQ qPCR SYBR Green Master Mix (Vazyme, China) following the manufacturer’s protocols. The relative mRNA expression was determined using the 2^−ΔΔCt^ method, and the internal reference Glyceraldehyde 3-phosphate dehydrogenase (GAPDH) was used. Table S1 lists the primer sequences used for the qRT-PCR. All the primers were purchased from Tsingke (Wuhan, China).

### 2.11. Statistical Analysis

According to the methods described above, all statistical analyses were performed using the R software (version 4.1.1), Perl language packages, and GraphPad Prism software (version 8). All statistical results with *p* < 0.05 were considered statistically significant.

## 3. Results

### 3.1. Construction of Differentially Expressed UbRGPs and Identification of LUAD Subtypes

**Figure 2:**
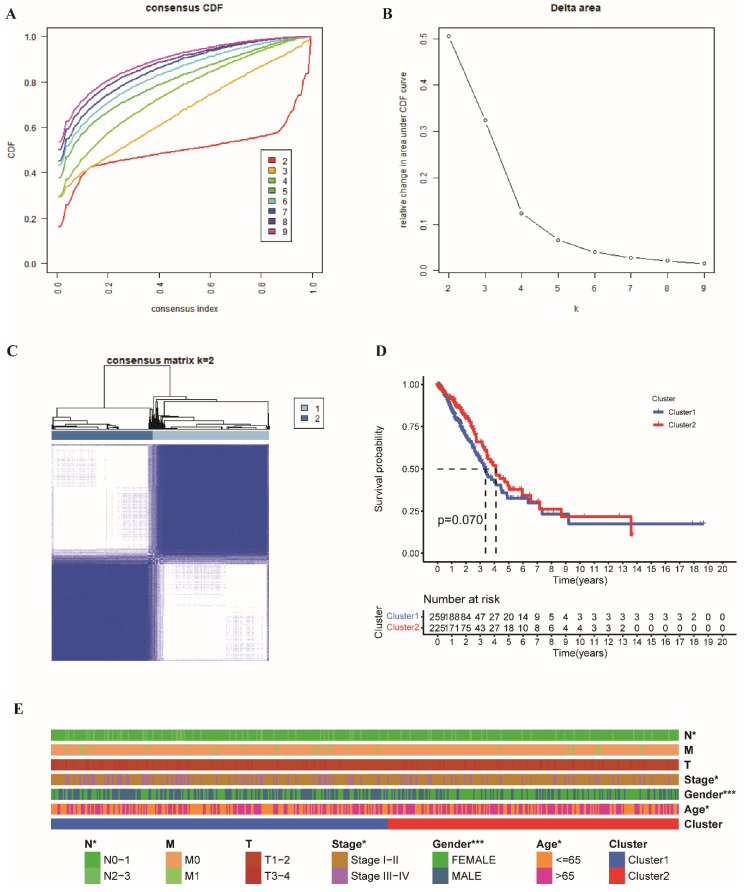
Unsupervised consensus clustering analysis of the patients with lung adenocarcinoma (LUAD) from The Cancer Genome Atlas (TCGA) database based on ubiquitin-related gene pairs (UbRGPs). (**A**) Consensus CDF at k = 2 − 9. (**B**) Delta area under the cumulative distribution function (CDF) curves for different clusters from k = 2 − 9. (**C**) Consensus matrix when k = 2. (**D**) The overall survival (OS) probability of the patients in the two clusters. (**E**) The distribution of clinical characteristics, including N, M, T, and TNM stages; gender; and age of the two clusters. (* *p* < 0.05; *** *p* < 0.001).

A total of 633 ubiquitin-related genes (UbRGs) were detected in the TCGA and GSE13213 datasets used in this study. The differentially expressed UbRGs were selected according to the screening criteria of FDR <0.05 and |log2 FC| > 1. The expression and characteristics of differentially expressed UbRGs are shown in Figure S1. Inspired by the study of Li et.al, ubiquitin-related gene pairs (UbRGPs) were constructed to avoid the bias introduced by different platform measurements [21]. Among the 96 differentially expressed UbRGs in tumor tissues, 624 valid UbRGPs were defined by an iteration loop and 0-or-1 expression matrix. Next, the expression profiles of these 624 UbRGPs were used for consensus clustering analysis of LUAD. A total of 484 patients with LUAD matched with clinicopathological features from the TCGA database were included. According to the consensus CDF curves (Figure 2A), the relative change in the area under the CDF curves (Figure 2B), and the consensus matrix (Figure 2C), the best division (k = 2) was the best cluster. The 484 patients with LUAD were divided into two subtypes, and the patients who belonged to Cluster 1 (*n* = 259) had poorer outcomes than those in Cluster 2 (*n* = 225; *p* = 0.07, Figure 2D). The clinicopathological features of UbRGPs in Clusters 1 and 2 are shown in a heatmap (Figure 2E). The chi-square analysis showed that N stage, TNM stage status, gender, and age were different between Clusters 1 and 2. The proportion of patients with a lower tumor stage (stage I–II) and lower lymph node metastasis (N0–N1) was higher in Cluster 2 than that in Cluster 1 (Figure 2E). There were no differences in the M and T stages between the two clusters. GSEA was further analyzed (Figure S2). Gene Ontology (GO) biological process showed that chromosome segregation and organelle fission were enriched in Cluster 1 (Figure S2A), whereas antigen processing and presentation, αβT cell differentiation and activation, and activation of immune response were enriched in Cluster 2 (Figure S2B). Kyoto Encyclopedia of Genes and Genomes (KEGG) pathway analysis demonstrated that DNA replication and cell cycle were enriched in Cluster 1 (Figure S2C), while Cluster 2 was involved in cell adhesion molecules and chemokine signaling pathways (such as IL-6_JAK_STAT3 signaling) (Figure S2D). These results suggested there are differences between the two clusters.

### 3.2. Tumor Microenvironment Characterization in Two Subtypes

**Figure 3:**
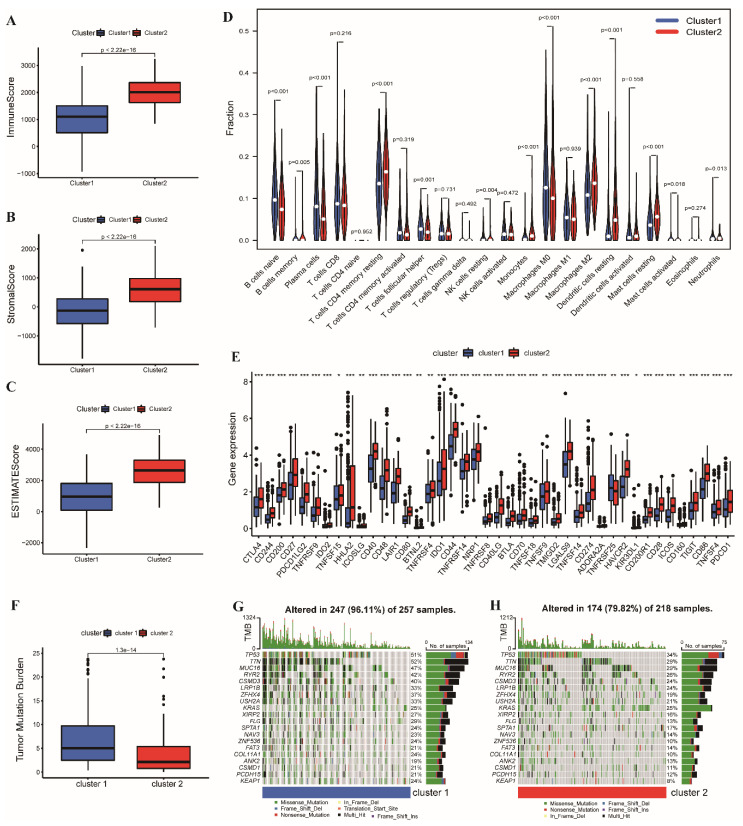
The tumor microenvironment (TME) characteristic of the patients between the two clusters in the TCGA cohort. The distributions of the (**A**) immune score, (**B**) stromal score, and (**C**) ESTIMATE score between the two clusters. (**D**) The fraction of each infiltrating immunocyte in Clusters 1 and 2 by CIBERSORT. (**E**) The boxplot of the 42 differentially expressed immune checkpoint inhibitor (ICI)-related genes in the two clusters. (**F**)The boxplot of tumor mutation burden (TMB) of the two clusters. (**G**,**H**) The waterfall plots of the 20 most frequently mutated genes in the two clusters. (2.22e−16 = 2.22 × 10^−16^; * *p* < 0.05; ** *p* < 0.01; *** *p* < 0.001).

GSEA results indicated that some chemokine signaling pathways and adaptive immune-related processes were enriched in Cluster 2 (Figure S2). First, we estimated TME scores in the TCGA cohort using the ESTIMATE algorithm. The results indicated that the stromal, immune, and ESTIMATE scores of samples in Cluster 2 were higher than those in Cluster 1 (Figure 3A–C). Immune cell infiltration was further analyzed. The percentages of B cells, plasma cells, follicular helper T cells, resting memory CD4 + T cells, resting natural killer cells, M0 and M2 macrophages, resting dendritic cells, mast cells, and neutrophil infiltration were different between Clusters 1 and 2 (Figure 3D). In addition, we compared 47 immune checkpoint inhibitor (ICI) related genes retrieved from published literature in the two clusters. The results indicated that 42 ICI-related genes, including CTLA4, PD-L2 (PDCD1LG2), and PD-L1 (CD274) were upregulated in Cluster 2 (Figure 3E). We found that the relative probability of response to CTLA4_positive_/PD-1_negative_ treatment in Cluster 1 was higher than that in Cluster 2 based on the IPS scoring strategy (Figure S3). There was no statistical difference in the relative response probability of CTLA4_negative_/PD-1_positive_, CTLA4_positive_/PD-1_positive_, and CTLA4_negative_/PD-1_negative_ treatment between the two clusters.

Moreover, we investigated the difference in somatic mutations using the maftools package. As presented in Figure 3F, the TMB was higher in Cluster 1. The mutation frequencies of the top 20 mutant genes (*TP53* and *KRAS*) in Cluster 1 were higher, and the altered proportion of patients was 96.11% and 79.82%, respectively. Figure 3G,H shows the top 20 most frequently mutated genes in Clusters 1 and 2.

### 3.3. Establishment and Evaluation of Prognostic Signature

**Figure 4:**
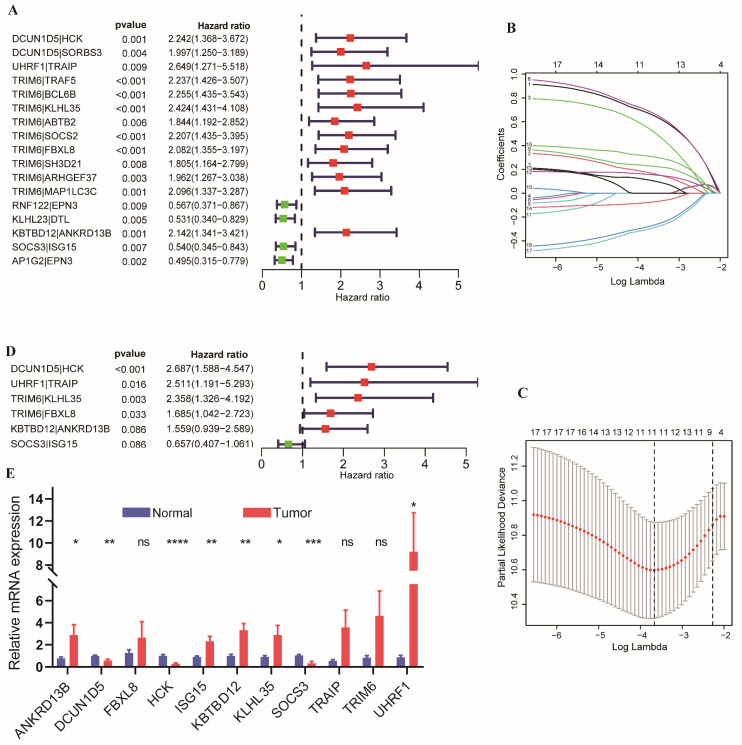
The prognostic signature of patients with LUAD was established based on UbRGPs in the TCGA training set. (**A**) Forest map of the 17 UbRGPs related to OS. (**B**) The distribution of the LASSO coefficient. (**C**) Selection of the 11 optimal parameters when Log Lambda = −3.7 in the LASSO model. (**D**) The forest map showed the six UbRGPs, which were used to establish the prognostic signature. (**E**) The relative mRNA expression levels of 11 UbRG genes in LUAD samples from Wuhan Union Hospital by qRT-PCR. Data are presented as the mean ± standard error of the mean (SEM). (* *p* < 0.05; ** *p* < 0.01; *** *p* < 0.001; **** *p* < 0.0001; ns, *p* ≥ 0.05).

The patients in the TCGA cohort were randomly separated into testing (*n* = 242) and training (*n* = 242) sets. Considering the criterion of *p* < 0.01, 17 UbRGPs were correlated with OS in the training set. The forest map (Figure 4A) revealed the 95% confidence interval (CI) and hazard ratio (HR) of each prognosis-related UbRGP, indicating that most of these pairs were risk factors, and the protective factors, including four UbRGPs. The above-mentioned 17 prognosis-related UbRGPs were analyzed for further LASSO analysis in the TCGA training set, and 11 UbRGPs were selected (Figure 4B,C) for the stepwise multivariate Cox analysis. Eventually, six UbRGPs were used in the subsequent prognostic signature construction, and the details of the six UbRGPs are shown in Table 2. The forest map indicated that the *DCUN1D5*|*HCK*, *UHRF1*|*TRAIP*, *TRIM6*|*KLHL35*, *TRIM6*|*FBXL8*, and *KBTBD12*|*ANKRD13B* pairs were risk factors with the HR > 1, and only *SOCS3*|*ISG15* was a protective factor and HR < 1 (Figure 4D). The expression of the 11 UbRGs in cancer and para-cancerous normal lung samples of patients with LUAD was detected using qRT-PCR. As shown in Figure 4E, *ANKRD13B*, *ISG15*, *KBTBD12*, *KLHL35,* and *UHRF1* were upregulated in tumor samples; *DCUN1D5*, *HCK,* and *SOCS3* were downregulated in tumor than in normal samples.

**Figure 5:**
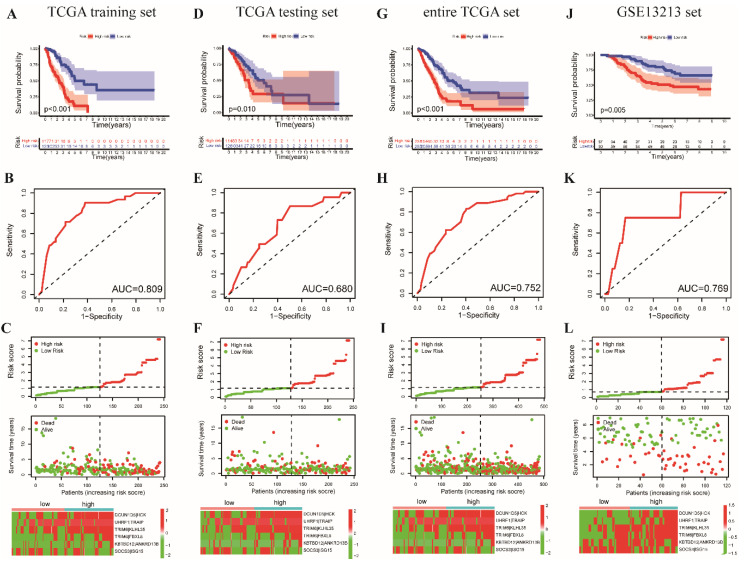
The survival curves of patients with LAUD in the low- and high-risk groups in the (**A**) training, (**D**) testing, (**G**) TCGA, and (**J**) GSE13213 sets. The AUC was predicted for 1-year survival prediction in the (**B**) training, (**E**) testing, (**H**) TCGA, and (**K**) GSE13213 sets. The risk score, survival status distribution, and heatmap of the expression of the 6 UbRGPs in patients in the (**C**) training, (**F**) testing, (**I**) TCGA, and (**L**) GSE13213 sets.

The risk score was calculated in the multivariate Cox proportional hazard model. Based on the median risk score, the patients with LUAD in the set of training were assigned to the low- (*n* = 125) and high-risk (*n* = 117) groups. Patients survived longer in the low-risk group (Figure 5A, *p* < 0.001). The area under the ROC curve (AUC) in the training set was 0.809 (Figure 5B) for one-year survival prediction. Figure 5C shows a scatter diagram of the risk score, survival status distribution, and heatmap of each patient in the training set. Furthermore, we used the testing and the entire TCGA dataset for internal validation. Patients showed better clinical outcomes in the low-risk group in the testing set (Figure 5D) and the entire TCGA dataset (Figure 5G, *p* < 0.001). The AUC value at one year for the testing set was 0.680 (Figure 5E). Similarly, the AUC of the TCGA dataset was 0.752 (Figure 5H). The scatter diagram shows the distribution of risk scores, survival status, and heatmap of patients in the testing set (Figure 5F) and entire the TCGA dataset (Figure 5I).

To confirm the performance of the UbRGP signature for survival prediction in different datasets, we employed another independent dataset, GSE13213, for external validation. Similar results were observed, with a lower probability of survival in the high-risk group and better prognosis in the low-risk group (Figure 5J). Moreover, the AUC value of the 1-year survival ROC curve for the GSE13213 dataset was 0.769 (Figure 5K). The distribution of the risk score, survival status, and heatmap in the GSE13213 dataset is shown in Figure 5L. In conclusion, the above results illustrate that the six UbRGPs signature can predict the survival probability of patients with LUAD in both internal and external validation datasets.

### 3.4. Correlation between the Clinical Features and Risk Score

**Figure 6:**
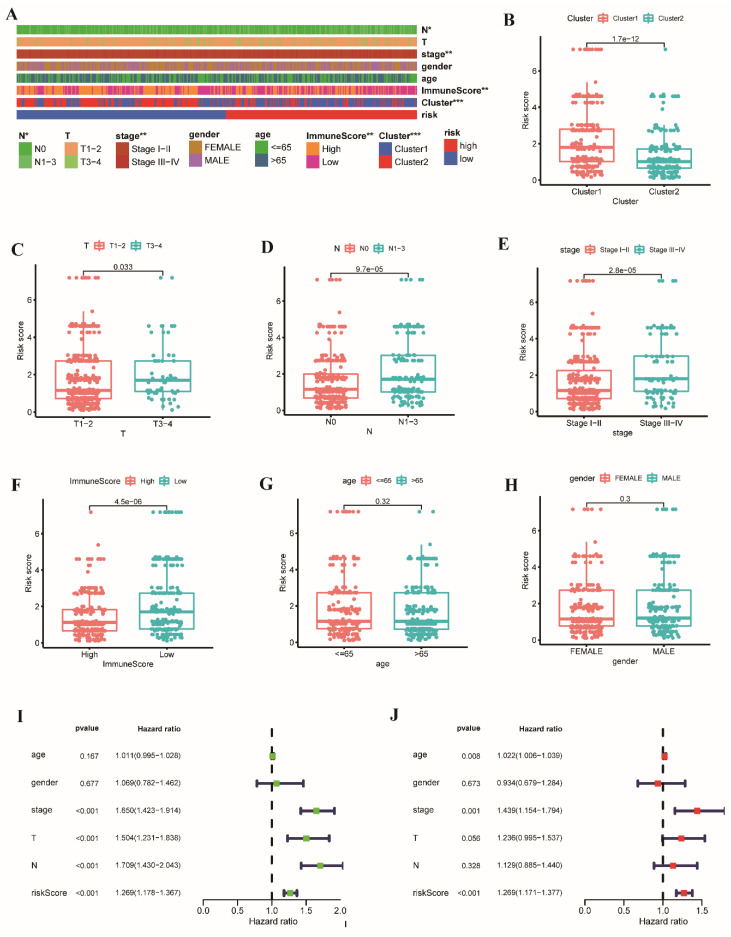
Correlation analysis between the clinical features and risk score of patients with LUAD in the TCGA cohort. (**A**) The heatmap of the clinical features of patients in the low- and high-risk groups. The relationship between the clinical parameters and risk score is shown in the boxplot, including (**B**) cluster, (**C**) T stage, (**D**) N stage, (**E**) TNM stage, (**F**) immune score, (**G**) age, and (**H**) gender of patients. (**I**) Univariate and (**J**) multivariate Cox analysis of signature-based risk score and other clinical features. (1.7e−12 = 1.7 × 10^−12^; * *p* < 0.05; ** *p* < 0.01; *** *p* < 0.001).

As illustrated in Figure 6A, there were differences between the low- and high-risk groups in T, N, and TNM stages; immune score; and cluster subtype. Subsequently, the correlation between the signature-based risk scores and clinical parameters (age, gender, immune score, cluster, and T, N, and TNM stages) was analyzed in the TCGA cohort. We observed that the risk scores of patients in Cluster 1 were higher than in Cluster 2 (Figure 6B; *p* < 0.001). Patients with stages T3–4 had higher risk scores than those with stages T1–2 (Figure 6C, *p* = 0.033), and patients with stages N1–3 had higher risk scores than those with stage N0 (Figure 6D, *p* < 0.001), and patients with stages III–IV had higher risk scores than patients with stages I–II (Figure 6E, *p* < 0.001). Patients with high immune scores had lower risk scores than patients with low immune scores (Figure 6F; *p* < 0.001). However, there were no differences in age (Figure 6G, *p* = 0.32) or gender (Figure 6H, *p* = 0.3).

We performed univariate and multivariate Cox regression analyses to investigate the function of risk score. The results revealed that the risk score was associated with OS in univariate Cox regression analysis (HR = 1.269, 95% CI = 1.178–1.367, *p* < 0.001, Figure 6I). Multivariate analysis also demonstrated that the risk score was an independent prognostic indicator (HR = 1.269, 95% CI = 1.171–1.377, *p* < 0.001, Figure 6J). Furthermore, to validate the applicability and stability of the UbRGP prognostic signature, we applied survival analysis of subtypes in the two groups according to age, gender, T, N, and TNM stages. In all subgroups, the clinical outcomes of patients were better in the low-risk group (Figure S4, *p* < 0.05). 

### 3.5. Establishment and Evaluation of Clinical Nomogram and Calibration Curves

**Figure 7:**
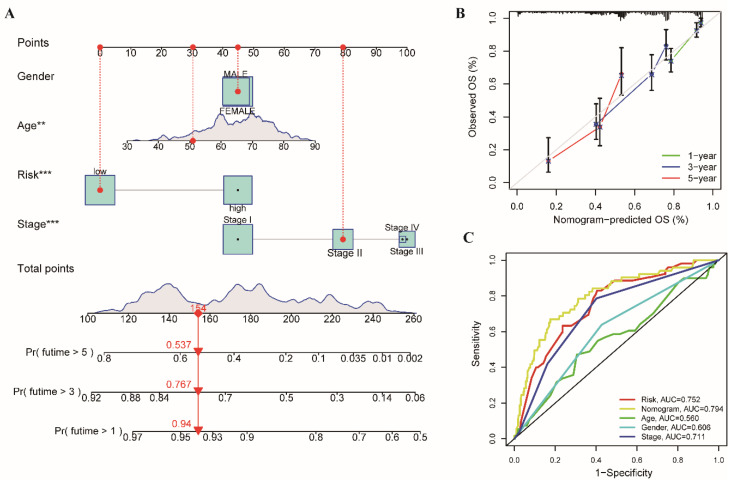
Establishment and evaluation of the nomogram of patients with LUAD in the TCGA cohort. (**A**) The survival prediction of patients with risk groups and clinical features was shown in the nomogram. (**B**) The calibration curves of the nomogram for the predicted OS probability and observed OS at 1-year, 3-year, and 5-year. (**C**) ROC curves of the nomogram. (** *p* < 0.01; *** *p* < 0.001).

Furthermore, we estimated the 1-, 3-, and 5-year individual survival probabilities of patients by constructing a nomogram (Figure 7A). A calibration chart of the nomogram was used to confirm the accuracy and sensitivity of the prediction, and the calibration curve showed that the actual and predicted survival rates matched well at 1-, 3-, and 5-year intervals (Figure 7B). The ROC curve showed that the 1-year AUC of the nomogram was 0.794, outperforming other clinical features (risk, AUC = 0.752; age, AUC = 0.560; gender, AUC = 0.606; stage, AUC = 0.711; Figure 7C). The results indicated that the nomogram had high accuracy in predicting OS.

### 3.6. TME Cell Infiltration Characteristics and Somatic Mutation

**Figure 8:**
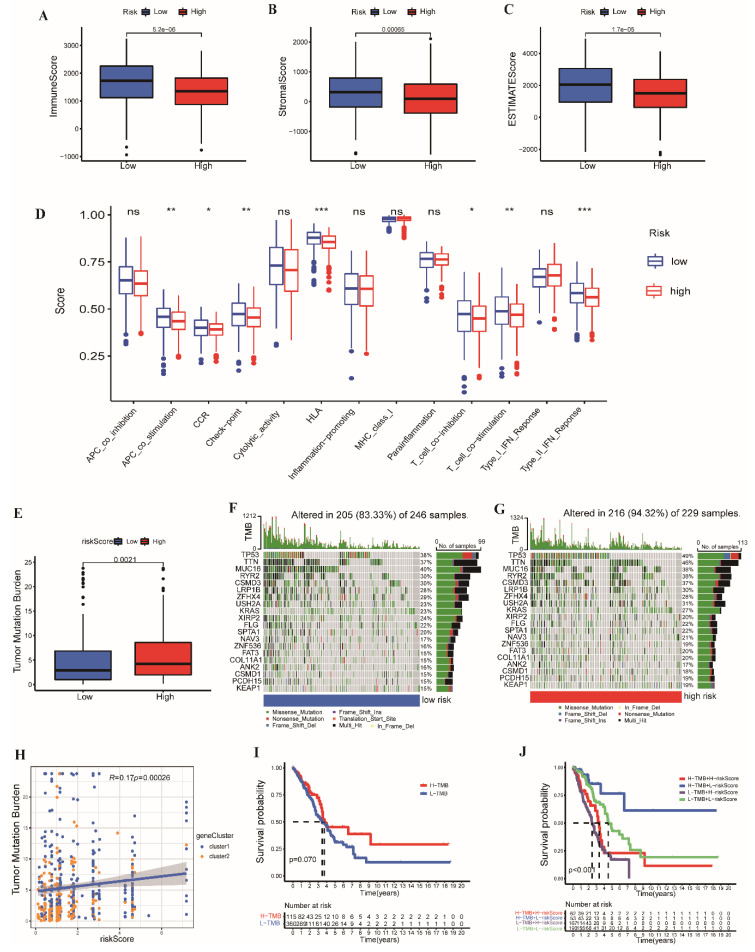
The distributions of the (**A**) immune score, (**B**) stromal score, and (**C**) ESTIMATE score of the low- and high-risk groups. (**D**) The proportion of the 13 immune-related pathways of the two groups. (**E**) The TMB boxplot of the two groups. (**F**,**G**) The waterfall plots of the top 20 most frequently mutated genes in low- and high-risk groups. (**H**) The correlation analysis between the TMB and risk score based on UbRGPs. Survival curves for patients grouped by (**I**) TMB alone (*p* = 0.07), and (**J**) the combination of the risk score and TMB (*p* < 0.001). (5.2e−06 = 5.2 × 10^−6^; * *p* < 0.05; ** *p* < 0.01; *** *p* < 0.001; ns, *p* ≥ 0.05).

Subsequently, we estimated the TME scores between the low- and high-risk groups. The low-risk group had higher stromal, immune, and ESTIMATE scores (Figure 8A–C). Considering the significant differences in the immune scores between the two groups, we further mined the immune cell infiltration. We evaluated the immunocyte components in LUAD tissues between the two groups. A heatmap of various immune cell components based on CIBERSORT-ABS, CIBERSORT, EPIC, MCPCOUNTER, QUANTISEQ, TIMER, and xCELL is shown in Figure S5. Furthermore, we compared the enrichment scores of the immune-related pathways in the two groups. We found the low-risk group with an abundance of immune-related pathways (Figure 8D). The results exhibited distinct characteristics of immunocyte infiltration in two groups, and there was a relationship between tumor immune microenvironment and risk score.

The somatic mutations in the low- and high-risk groups were analyzed. Patients had a more extensive TMB in the high-risk group (Figure 8E). As shown in Figure 8F,G, the mutation frequency of each gene was higher in the high-risk group. The altered proportion of samples in the high-risk group was 94.332%, whereas that in the low-risk group was 83.33%. In addition, the risk score exhibited a positive correlation with TMB. With an increase in the risk score, the TMB increased (Figure 8H). The patients with high TMB had a shorter OS (Figure 8I; *p* = 0.07). We further combined the risk score and TMB for survival analysis. According to Figure 8J, there were differences among the four groups (*p* < 0.001).

### 3.7. Relationship between Drug Sensitivity and Prognostic Signature

To further investigate the performance in predicting the response of patients with LUAD in the low- and high-risk groups to ICI immunotherapy by the prognostic signature, we compared the expression levels of 47 ICI-related biomarkers between the two groups. The results revealed that the expression of 24 ICI-related genes (such as *ICOS*, *CD28*, and *CD80/86*) was upregulated in the low-risk group (Figure S6). The most common ICI-related genes, *PD1* and *CTLA4*, did not differ between the two groups. In addition, the IPS scoring results demonstrated that patients in two groups had the same relative probability of response to anti-CALT4 and/or anti-PD-1 (data not shown). The data suggest that patients may respond better to novel immunotherapies in the low-risk group.

**Figure 9:**
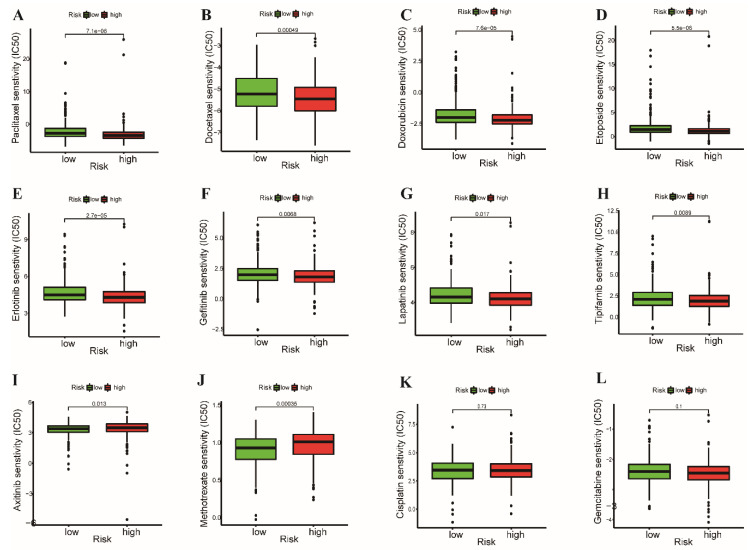
Prediction of the drug sensitivity (IC_50_) of (**A**) paclitaxel, (**B**) docetaxel, (**C**) doxorubicin, (**D**) etoposide, (**E**) erlotinib, (**F**) gefitinib, (**G**) lapatinib, (**H**) tipifarnib, (**I**) axitinib, (**J**) methotrexate, (**K**) cisplatin, and (**L**) gemcitabine. *p* < 0.05 is considered statistically significant. (7.1e−06 = 7.1 × 10^−6^).

We further explored the correlation between drug sensitivity of chemo- and targeted therapy and the prognostic signature. Patients in the high-risk group showed higher sensitivity (lower IC_50_) to paclitaxel, docetaxel, doxorubicin, etoposide, erlotinib, gefitinib, lapatinib, and tipifarnib (Figure 9A–H). Patients showed higher resistance (higher IC_50_) to axitinib and methotrexate in the high-risk group (Figure 9I,J). However, there was no difference in the sensitivity to gemcitabine and cisplatin between the two groups (Figure 9K,L). These findings suggest that the prognostic signature has great potential in predicting the sensitivity of immune-, chemo-, and targeted therapy, which can guide clinical treatment choices and achieve satisfactory clinical outcomes.

## 4. Discussion

LUAD is a common pathological type of lung cancer worldwide. It is an urgent need to identify the new therapeutic targets and prognostic biomarkers. In this study, we investigated the characteristics of UbRGPs, analyzed the relationship between UbRGPs and TME, and predicted the response in patients with LUAD to immune-, chemo-, and targeted therapy.

First, we identified 633 differentially expressed UbRGs by comparing gene expression between LUAD cancer samples and para-cancerous normal lung samples in the TCGA cohort. Differentially expressed UbRGs were mainly involved in ubiquitin-mediated proteolysis, as indicated by GO and KEGG analysis. As demonstrated by Li et al. [21], we constructed differentially expressed UbRGPs, which were the comparative ranking of UbRG expression in tumor tissues. According to the expression matrix of the 624 UbRGPs, patients with LUAD were separated into two clusters with different survival outcomes, clinicopathological features, KEGG enrichment pathways, immunocyte infiltration, ICI-related gene expression, and somatic mutation. Specifically, αβT cell activation and differentiation, antigen processing and presentation, activation of the immune response, and chemokine signaling pathways were more enriched in Cluster 2 than in Cluster 1. Cluster 2 expressed more ICI-related genes and had a lower TMB than Cluster 1. Nikolaj et al. demonstrated that the immunotherapeutic responses vary with the tumor mutations in different subtypes of patients with LUAD [40,41]. In summary, we considered that UbRGPs were associated with cancer progression and clinical prognosis of patients with LUAD.

Next, UbRGPs were used to construct a reliable prognostic signature for patients with LUAD and to avoid expression differences between different platforms, such as microarray, RT-PCR, and RNA sequencing. We established a prognostic signature of six UbRGPs composed of 11 UbRGs (*ANKRD13B*, *DCUN1D5*, *FBXL8*, *HCK*, *ISG15*, *KBTBD12*, *KLHL35*, *SOCS3*, *TRAIP*, *TRIM6*, and *UHRF1*), which performed efficiently in stratifying the low- and high-risk patients with LUAD from the GSE13213 and TCGA and cohorts.

Some of these 11 UbRGs are associated with the initiation and progression of lung cancer. Overexpression of *DCUN1D5* was associated with reduced disease-specific survival in oral and lung squamous cell carcinomas [42] and breast cancer [43]. Hematopoietic cell kinase (*HCK*) overactivation is related to LUAD [44] and several types of leukemia [45]. Inhibition of *HCK* activity suppresses myeloid cell-mediated colon cancer progression [46]. *ISG15* inhibited lung cancer cell growth by promoting its ubiquitin E3 ligase activity [47]. Compared with normal lung tissues, 25% of NSCLC patients had high expression of *ISG15* in tumors and were more sensitive to topotecan therapy [48]. The expressions of *ISG15* were upregulated in esophageal squamous cancer [49] and gastric cancer [50]. The downregulated expression of *KLHL35* was positively correlated with poor prognosis in patients with LUAD [51]. Methylation of *KLHL35* was significantly higher in cancer tissue than in noncancerous tissue of liver cancer [52] and renal cell carcinoma [53]. The decreased expression of *SOCS3* promoted the proliferation, migration, invasion, anti-anoikis apoptosis, and gemcitabine resistance of A549 cells by negatively regulating the JAK/STAT signaling pathway [54]. Downregulation of *SOCS3* expression was associated with the TNM stage and poor prognosis of patients with lung cancer [55,56]. *TRAIP* was a new regulator in DNA damage response and was associated with cancer development in LUAD [57,58]. The elevated expression of *TRAIP* in cancer samples promoted tumor metastasis and poor survival in patients with lung cancer [59]. *TRIAP* ubiquitin ligase promoted the sensitivity of breast cancer cells to camptothecin [60], and the malignant behaviors in liver cancer [61] and osteosarcoma cells [62]. The overexpression of *TRIM6* in LUAD tissues indicated a poor prognosis [63]. *TRIM6* accelerated the growth and metastasis of breast cancer [64] and colorectal cancer [65,66]. UHRF1 is a novel diagnostic marker of lung cancer, *UHRF1* controls the cell cycle by silencing tumor suppressor genes, and the overexpressed *UHRF1* was related to the poor survival of NSCLC patients [67,68,69]. Overexpression of *UHRF1* rescued double-strand break repair, and depletion of *UHRF1* reduced the chemosensitivity of KRAS mutant lung cancer cells [70]. The other three genes had not been previously reported in lung cancer but are known to be expressed in other tumors. *ANKRD13B* was overexpressed in tumor tissues compared with normal renal tissue, and the higher expression indicated that patients with renal cell carcinoma had an advanced clinical stage and low OS [71,72]. *FBXL8* was significantly upregulated in human breast carcinoma tissues [73,74]. *KBTBD12* expression was downregulated in colorectal adenoma compared with hyperplastic polyps [75]. The function of *ANKRD13B*, *FBXL8*, and *KBTBD12* in the carcinogenesis and progression of lung cancer need to be further investigated.

The crosstalk between tumor cells and the microenvironment affects the tumorigenesis and development of tumors [76]. TME is composed of stromal, immune, and tumor cells, which collectively interact with each other and affect the sensitivity to immunotherapy [77]. In our study, patients in the low-risk group exhibited higher immune scores, stromal scores, estimated scores, and lower TMB, all of which implied that the tumor purity was lower and might benefit from immunotherapy than those in the high-risk group [78]. Moreover, the higher expression of the ICI-related genes (such as *ICOS*) was observed in the low-risk group, indicating that patients in the low-risk group might have a superior response to the ICI and cancer vaccines than those in the high-risk group. However, patients in the low-risk group were more resistant to chemotherapeutic drugs (such as etoposide, docetaxel, doxorubicin, and paclitaxel) and targeted therapeutic drugs (such as gefitinib, erlotinib, lapatinib, and tipifarnib). Nevertheless, patients in the low-risk group exhibited a higher sensitivity to axitinib, metformin, and methotrexate. The prognostic signature based on the six UbRGPs has great potential in assisting the clinical therapeutic options for LUAD.

This study had some limitations. First, we employed gene pairs to construct a prognostic signature to avoid bias from data sources and validated it using internal and external datasets; however, there is still inevitable bias due to the genetic heterogeneity of tumors. Second, more experiments need to be carried out to reveal the underlying specific mechanisms. Nevertheless, our study can stratify the risk of LUAD and guide treatment options for patients with LUAD in clinical practice.

## 5. Conclusions

To date, this is the first study to assess the expression characteristics of UbRGPs in LUAD and to identify two LUAD subtypes with different TME according to the UbRGPs in tumor samples. Risk stratification based on the six UbRGPs prognostic signatures exhibited good performance for clinical applications in predicting the survival, TME status, and response of patients with LUAD to immuno-, chemo-, and targeted therapy.

## Figures and Tables

**Table 1:** Clinicopathological features of the patients in TCGA–LUAD cohort and GSE13213 cohort.

Covariates	Type	Entire TCGA	Testing	Training	*p* Value ^#^	GSE13213
		*n* = 484	*n* = 242	*n* = 242		*n* = 117
Age (%)	≤65	233 (48.14%)	119 (49.17%)	114 (47.11%)	0.7159	78 (66.67%)
	>65	251 (51.86%)	123 (50.83%)	128 (52.89%)		39 (33.33%)
Gender (%)	FEMALE	263 (54.34%)	132 (54.55%)	131 (54.13%)	1	57 (48.72%)
	MALE	221 (45.66%)	110 (45.45%)	111 (45.87%)		60 (51.28%)
Stage (%)	Stage I–II	384 (79.34%)	193 (79.75%)	191 (78.93%)	0.9106	92 (78.63%)
	Stage III–IV	100 (20.66%)	49 (20.25%)	51 (21.07%)		25 (21.37%)
T stage (%)	T1-2	422 (87.19%)	218 (90.08%)	204 (84.3%)	0.077	104 (88.89%)
	T3-4	62 (12.81%)	24 (9.92%)	38 (15.7%)		13 (11.11%)
N stage (%)	N0	322 (66.53%)	163 (67.36%)	159 (65.7%)	0.7726	87 (74.36%)
	N1-3	162 (33.47%)	79 (32.64%)	83 (34.3%)		30 (25.64%)
M stage (%)	M0	463 (95.66%)	233 (96.28%)	230 (95.04%)	0.6554	117 (100%)
	M1	21 (4.34%)	9 (3.72%)	12 (4.96%)		/

**#**. The difference between training and testing cohorts was calculated.

**Table 2:** The ubiquitin-related genes (UbRGs) used to construct the ubiquitin-related gene pairs (UbRGPs).

UbRG 1	Full Name	UbRG 2	Full Name	UbRGP	Coefficient
*DCUN1D5*	Defective in cullin neddylation 1 domain-containing 5	*HCK*	Hematopoietic cell kinase	*DCUN1D5*|*HCK*	0.98856
*UHRF1*	Ubiquitin-like with PHD and ring-finger domains 1	*TRAIP*	Tumor necrosis factor receptor-associated factor-interacting protein	*UHRF1*|*TRAIP*	0.920628
*TRIM6*	Tripartite motif-containing 6	*KLHL35*	Kelch-like family member 35	*TRIM6*|*KLHL35*	0.857858
*TRIM6*	Tripartite motif-containing 6	*FBXL8*	F-box and leucine-rich repeat protein 8	*TRIM6*|*FBXL8*	0.521508
*KBTBD12*	Kelch repeat and BTB domain-containing 12	*ANKRD13B*	Ankyrin repeat domain 13B	*KBTBD12*|*ANKRD13B*	0.443928
*SOCS3*	Suppressor of cytokine signaling 3	*ISG15*	ISG15 ubiquitin-like modifier	*SOCS3*|*ISG15*	−0.42018

## Data Availability

All the data are available from the public databases, including the TCGA database (https://portal.gdc.cancer.gov/, last accessed on 30 January 2022), GEO database (https://www.ncbi.nlm.nih.gov/geo/, last accessed on 20 December 2021), and iUUCD 2.0 database (http://iuucd.biocuckoo.org/, last accessed on 10 October 2021), and GSEA analysis (http://www.broadinstitute.org/gsea, last accessed on 24 March 2022).

## Supplementary Materials

The following supporting information can be downloaded at: https://www.mdpi.com/article/10.3390/cancers14143478/s1, Figure S1: Detection of differentially expressed ubiquitin-related genes (UbRGs); Figure S2: Gene-set enrichment analyses (GSEAs) for ubiquitin-related gene pairs (UbRGPs) of patients with LUAD in the TCGA cohort; Figure S3: The immunophenoscore (IPS) scoring scheme evaluated the difference in the potential response to immunotherapy of patients in Clusters 1 and 2; Figure S4: Kaplan–Meier survival curves of patients with LUAD from the TCGA cohort in 10 clinical stratified subgroups; Figure S5: The correlation of tumor environment with the risk score using 7 algorithms; Figure S6: The gene expression of the immune checkpoint inhibitor (ICI)-related genes in the two groups; Table S1: The primer sequences of 11 ubiquitin-related genes (UbRGs) were used in quantitative real-time polymerase chain reaction (qRT-PCR) analysis.

## Abbreviations

ANKRD13B	Ankyrin repeat domain 13B
CDF	Cumulative distribution function
CI	Confidence interval
DCUN1D5	Defective in cullin neddylation 1 domain-containing 5
FBXL8	F-box and leucine-rich repeat protein 8
FBXW7	F-box and WD repeat domain-containing 7
GAPDH	Glyceraldehyde 3-phosphate dehydrogenase
GO	Gene Ontology
GSEA	Gene-Set Enrichment Analysis
HCK	Hematopoietic cell kinase
HR	Hazard ratio
ICI	Immune checkpoint inhibitor
ISG15	ISG15 ubiquitin-like modifier
IPS	Immunophenoscore
KBTBD12	Kelch repeat and BTB domain-containing 12
KEGG	Kyoto Encyclopedia of Genes and Genomes
KLHL35	Kelch-like family member 35
LASSO	Least absolute shrinkage and selection operator
LUAD	Lung adenocarcinoma
NSCLC	Non-small cell lung cancer
OS	Overall survival
TCGA	The Cancer Genome Atlas
TMB	Tumor mutation burden
TME	Tumor microenvironment
TRAIP	Tumor necrosis factor receptor-associated factor-interacting protein
TRIM6	Tripartite motif-containing 6
UBE2	Ubiquitin binding enzyme E2
UbRGs	Ubiquitin-related genes
UbRGPs	Ubiquitin-related gene pairs
UHRF1	Ubiquitin-like with PHD and ring-finger domains 1
