# Supplementary material for: Identification of Ubiquitin-Related Gene-Pair Signatures for Predicting Tumor Microenvironment Infiltration and Drug Sensitivity of Lung Adenocarcinoma
Source: Cancers (Basel). 2022 Jul 18;14(14):3478. doi: 10.3390/cancers14143478 (PMC9317993; doi:10.3390/cancers14143478)
Supplement: Supplementary file 1 [file cancers-14-03478-s001.zip › cancers-1783604-supplementary.pdf]

Article

Identification of Ubiquitin-Related Gene-Pair Signatures for Predicting Tumor Microenvironment Infiltration and Drug Sensitivity of Lung Adenocarcinoma

Yumei Li [1,†], Lanfen An [2,3,†], Zhe Jia [1], Jingxia Li [1], E Zhou [1], Feng Wu [1], Zhengrong Yin [1], Wei Geng [1], Tingting Liao [1], Wenjing Xiao [1], Jingjing Deng [1], Wenjuan Chen [1], Minglei Li [1] and Yang Jin [1,*]

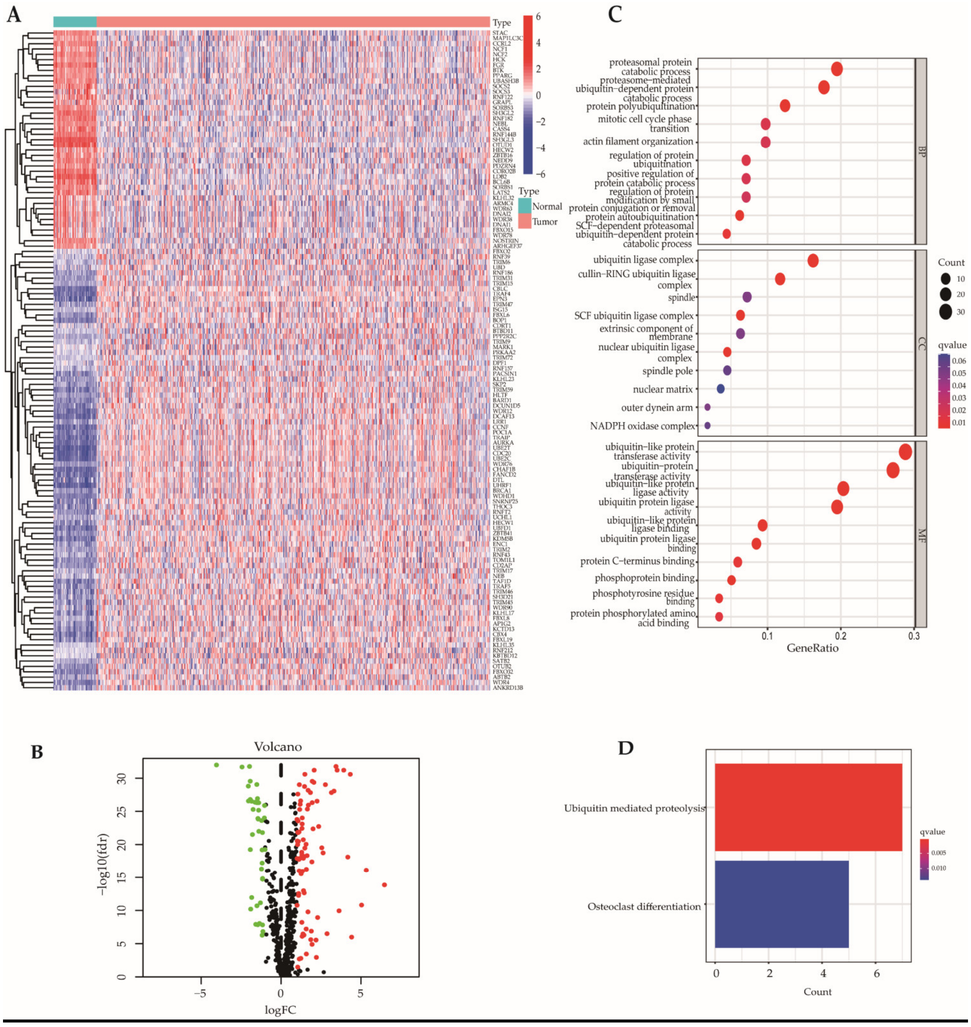

Figure S1. Detection of differentially expressed ubiquitin-related genes (UbRGs). (A) The top 100 differentially expressed UbRGs were presented in the heatmap. (B) The volcano plot of all identified differentially expressed UbRGs. The enrichment analysis of differentially expressed UbRGs by (C) the GO bubble, and (D) the KEGG pathway.

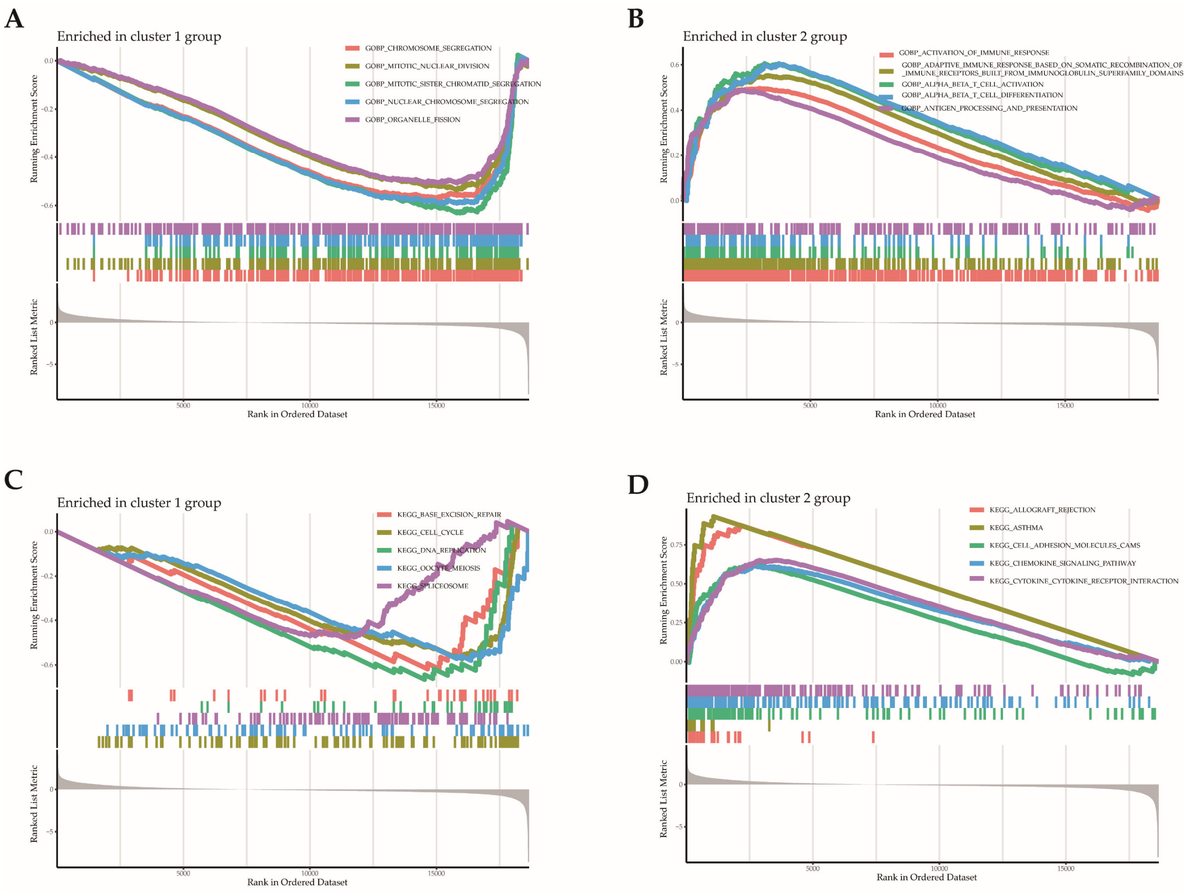

Figure S2. Gene set enrichment analyses (GSEAs) for ubiquitin-related gene pairs (UbRGPs) of patients with LUAD in the TCGA cohort. The GO terms were enriched in (A) Cluster 1 and (B) Cluster 2. The KEGG pathways were enriched in (C) Cluster 1 and (D) Cluster 2.

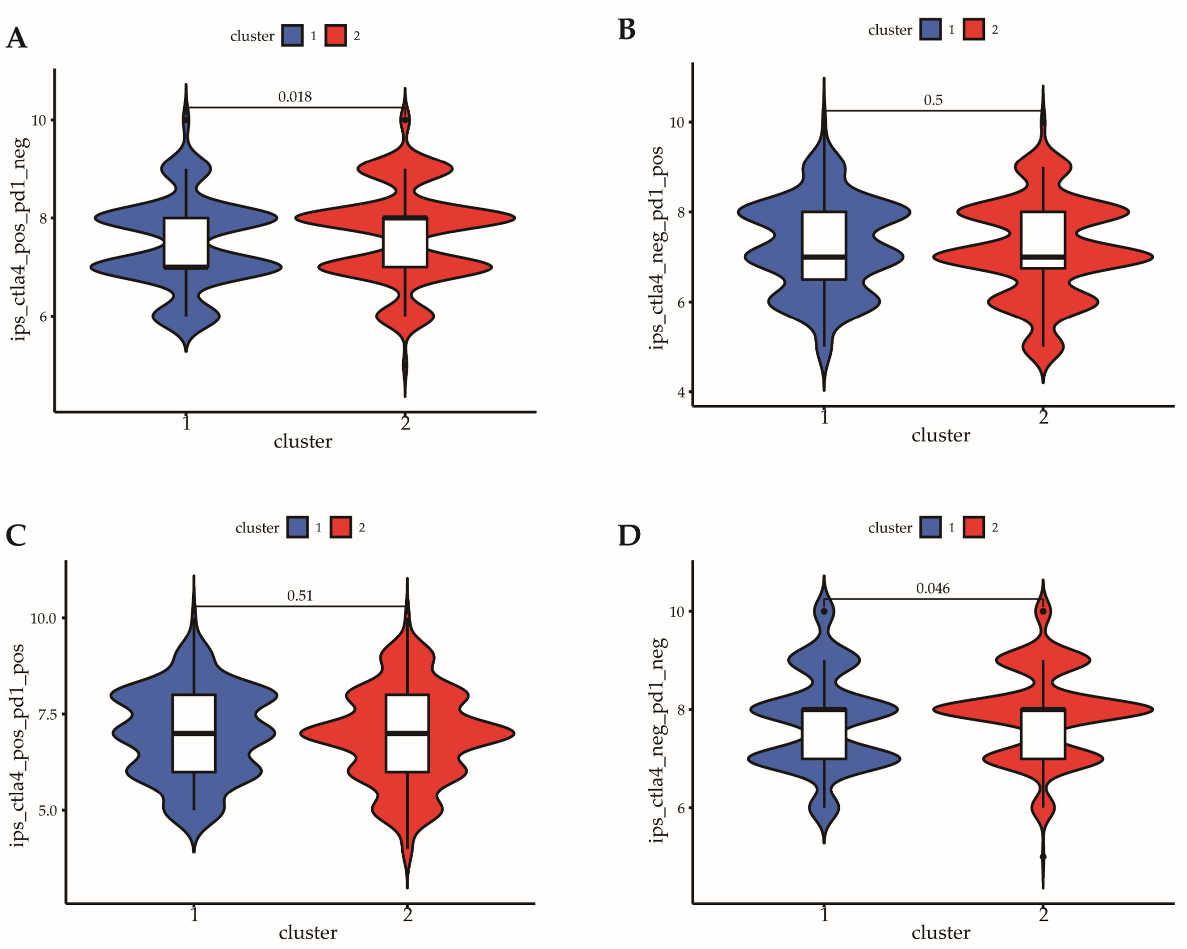

Figure S3. The immunophenoscore (IPS) scoring scheme evaluated the difference in the potential response to immunotherapy of patients in Clusters 1 and 2. $p < 0.05$ is considered statistically significant.

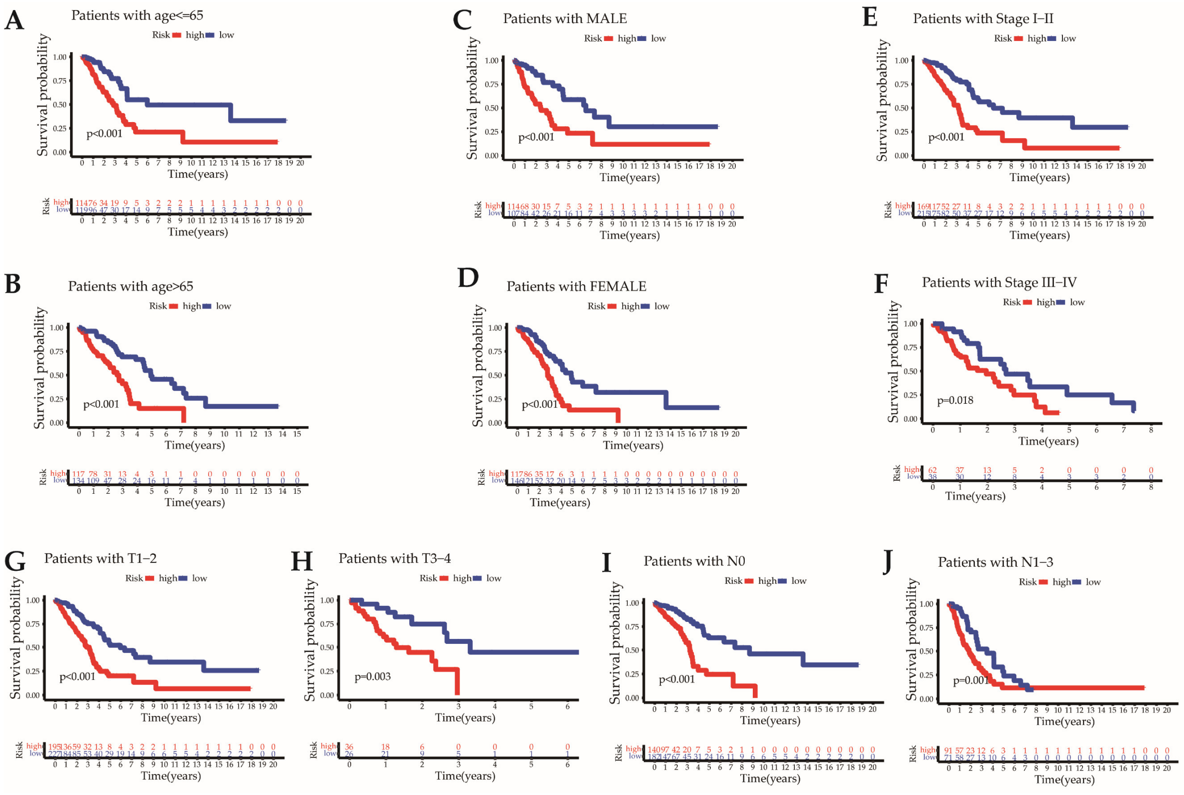

Figure S4. Kaplan–Meier survival curves of patients with LUAD from the TCGA cohort in 10 clinical stratified subgroups, including (A, B) age, (C, D) gender, (E, F) TNM stage, (G, H) T stage, (I, J) N stage. $p < 0.05$ is considered statistically significant.

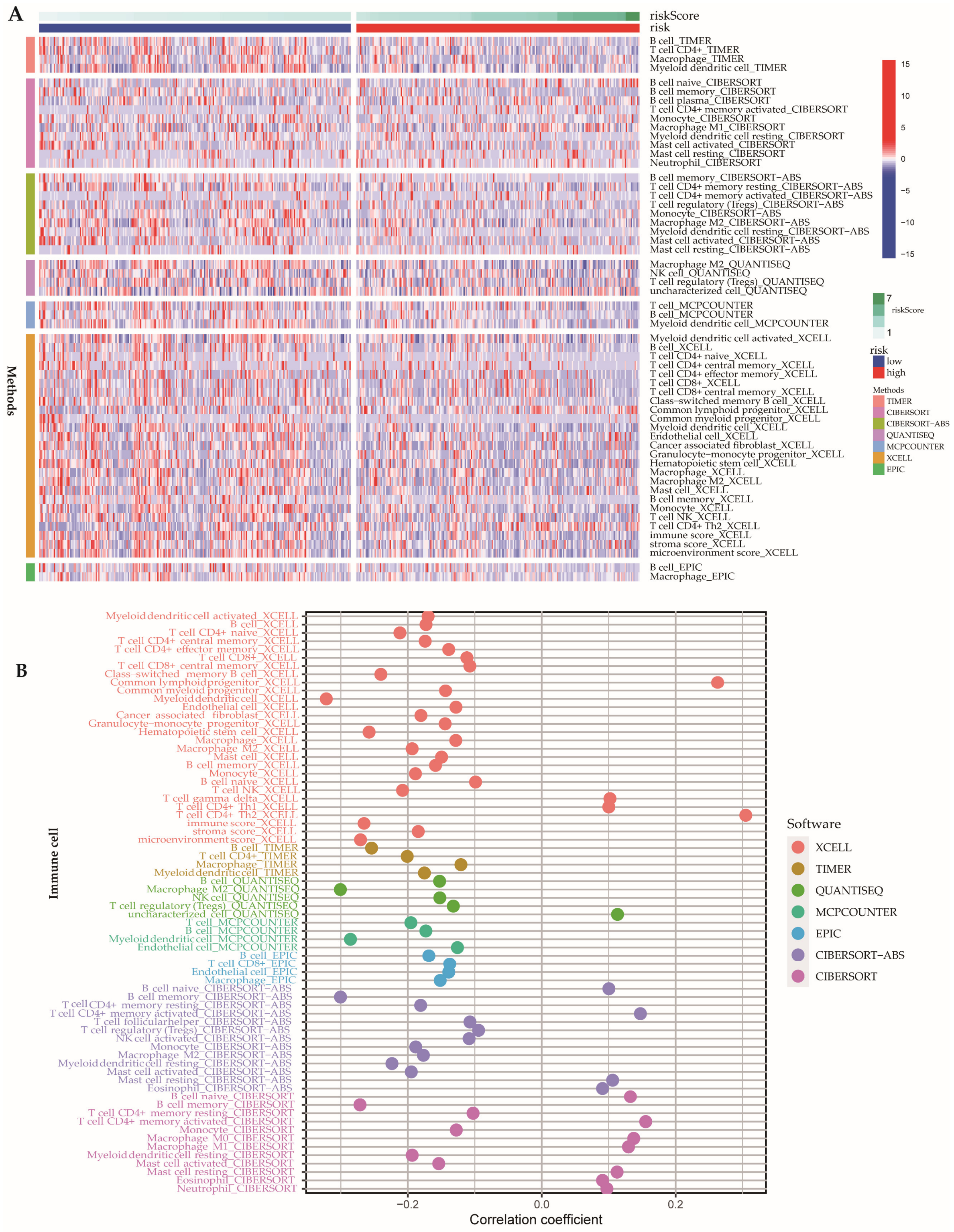

Figure S5. The correlation of tumor environment with the risk score using 7 algorithms. (A)The heatmap of stromal and immune cells in tumor environment of the low- and high-risk groups. (B) The Spearman correlation analysis between risk score and immune cells and stromal cells.

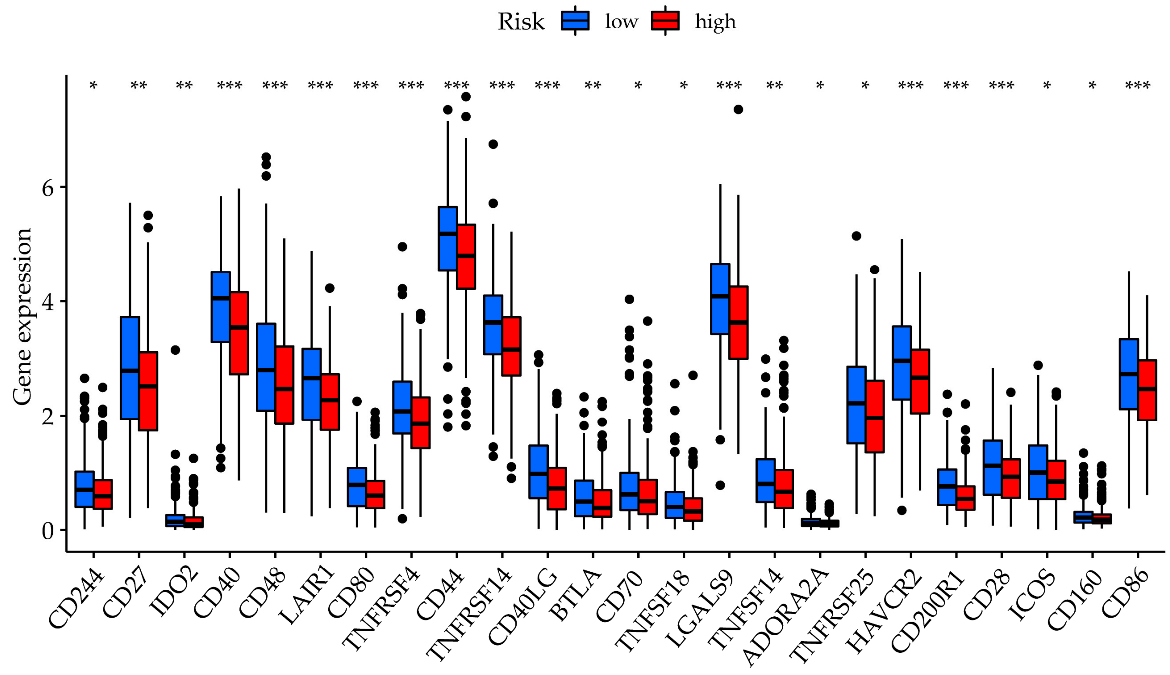

Figure S6. The gene expression of the immune checkpoint inhibitor (ICI)-related genes in the low- and high-risk groups. (*p < 0.05; **p < 0.01; ***p < 0.001).

Table S1. The primer sequences of 11 ubiquitin-related genes (UbRGs) were used in quantitative real-time polymerase chain reaction (qRT-PCR) analysis.

**Genes**	**Forward primer (5′-3′)**	**Reverse primer (5′-3′)**
ANKRD13B	TCACCACTCAGCTTGACACC	CCATCTCCGTCTTTTCACTGC
DCUN1D5	AGCAGAACAGTCCATGCTGA	CGTGGGAATTGAAAGGTTTGC
FBXL8	CGTGATTGGGCGACTTCTCT	GGGGGCAACAATCCCAAAAG
HCK	CCCTGTATGATTACGAGGCCA	CACTCCCCGGATTCCTCTAGG
ISG15	CGCAGATCACCCAGAAGATCG	TTCGTCGCATTTGTCCACCA
KBTBD12	ATGGACCACATGGATGCCTC	GTAAGCTCACCTCGGCAAAGT
KLHL35	CTTCTCACAGCGGTGTCTCG	GGTGAAGACCTTATCGGTGCTT
SOCS3	CAGCTCCAAGAGCGAGTACC	TGTCGCGGATCAGAAAGGTG
TRAIP	CGCTGGAAGAACGCAATGCTAC	GTGCTTGTTTGGTCTCATCCTGC
TRIM6	CATTTGCTGGCTTTGTGAGCGG	TTCCTGCTCCTCGTTCTTCAGC
UHRF1	GACAAGCAGCTCATGTGCGATG	AGTACCACCTCGCTGGCATCAT
GAPDH	GGAGCGAGATCCCTCCAAAAT	GGCTGTTGTCATACTTCTCATGG
